# Commentary: Totality of the Evidence Suggests Prenatal Cannabis Exposure Does Not Lead to Cognitive Impairments: A Systematic and Critical Review

**DOI:** 10.3389/fpsyg.2020.01891

**Published:** 2020-08-21

**Authors:** Kathleen H. Chaput, Catherine Lebel, Carly A. McMorris

**Affiliations:** ^1^Departments of Obstetrics and Gynecology and Community Health Sciences, Cumming School of Medicine, University of Calgary, Calgary, AB, Canada; ^2^Alberta Children's Hospital Research Institute, University of Calgary, Calgary, AB, Canada; ^3^Department of Radiology, Cumming School of Medicine, University of Calgary, Calgary, AB, Canada; ^4^School and Applied Child Psychology, Werklund School of Education, University of Calgary, Calgary, AB, Canada

**Keywords:** prenatal cannabis use, cognitive impairment, evidence, null findings, safety

Recent legalization of cannabis use in 40 American states and all Canadian provinces and territories has created an urgent need for evidence surrounding the implications of *in-utero* cannabis exposure for infant and child health outcomes, including cognitive functioning. A recently published systematic review by Torres et al. (2020) aimed to synthesize and critically appraise results from longitudinal studies examining the impact of prenatal cannabis exposure on multiple domains of cognitive functioning in individuals aged 0 to 22 years. The authors present a strong critical appraisal of the included literature and clearly highlight the limited evidence in this area. However, we have three primary concerns with the conclusions of the systematic review: (1) the statement “totality of the evidence” is misleading and cannot be interpreted without a meta-analysis; (2) the definition of “clinically significant” findings used to draw conclusions is limited; and (3) the lack of evidence for a harmful association between *in-utero* cannabis exposure and cognitive functioning should not be concluded as evidence for safety.

In their critical appraisal of the 45 studies included in the review, the authors rightly highlight serious limitations of previously published studies including problematic cognitive outcome measurement, lack of defined clinical significance of observed outcomes, limited measurement and control for confounding variables, limited measurement and quantification of cannabis exposure, and lack of adjustment for multiple statistical comparisons. We further offer the limitation that none of the studies included were adequately powered to detect clinically significant differences in the categorical outcome of abnormal vs. normal-range cognitive scores.

A meta-analysis was not conducted in this review due to significant heterogeneity of methods and outcome measurement in previous studies. However, the title of the review, and the interpretation of the results implies that studies were analytically combined. Without a meta-analysis, no stronger conclusions can be drawn from a group of individual underpowered and methodologically flawed studies than from each study individually (Haidich, 2010). Further, of the 45 papers included as separate studies, 80% (36 of 45) are derived from only three longitudinal cohorts (Fried and Watkinson, 1990; Day et al., 1994; Singer et al., 1999). The repeated analysis of the same individuals over time, rather than different individuals in 45 separate studies is not accounted for in the interpretation of the evidence. This, along with the reference to the “totality of the evidence” in the title, and the representation of results from 1001 statistical comparisons is therefore misleading.

Additionally, all three of these cohorts collected cannabis use data in the 1970s and 1980s. The potency of dried cannabis has increased significantly in the last 40+ years, as has the availability and usage of edible and topical cannabis products and high-potency oils and distillates (Mehmedic et al., 2010; ElSohly et al., 2016). The results of the review therefore cannot be generalized to prenatal cannabis exposure in the contemporary context.

More concerning is the fact that the finding of a lack of evidence of harm was interpreted as evidence for safety. Despite substantive methodological and statistical limitations identified in the included studies, indicating high risk for biased results, the authors conclude that “prenatal cannabis exposure does not lead to cognitive impairments.” The causal wording of this statement implies evidence for the safety of prenatal cannabis use in terms of offspring cognitive functioning, yet no such evidence exists. The conclusions in this review are centered on what the authors themselves present as likely biased results of the included studies. Without statistical synthesis of the individual studies in question, a conclusion of “no association” is not founded in the evidence, as none of the studies were powered adequately to detect a null-association with any meaningful precision (Button, 2016). Further, the authors' critique that, of the studies examined that had statistically significant findings, none are clinically meaningful, is also problematic. This argument rests largely on the use of normative ranges of outcome measures to define clinical significance. While the authors correctly assert that scores within the normal range do not necessarily indicate the presence of an intellectual or developmental disability, scores at the lower end of normal can still impact an individual's adaptive functioning, academic abilities, and long-term outcomes, such as employment, and independent living, especially in cases where lower scores persist over time. It is imperative to consider both statistical and clinical significance of study results, however, even a lack of observed clinically meaningful association cannot be equated to evidence of safety. Rather than evidence of safety, we propose that the results of this systematic review highlight that there is inadequate evidence to draw any conclusions about whether *in-utero* cannabis exposure is associated with cognitive functioning or not, especially in the contemporary context. Importantly, there is insufficient evidence to support a change in the current recommendations (Society of Obstetricians and Gynaecologists of Society of Obstetricians Gynaecologists of Canada, 2017; Braillon and Bewley, 2018) that individuals abstain from cannabis use during pregnancy and lactation.

The authors state that some existing policies pertaining to cannabis use in pregnancy are more harmful than prenatal cannabis use itself. We agree wholeheartedly that practices such as legal punitive action and child-apprehension are harmful to mothers and infants, but this remains true regardless of whether or not prenatal substance use is harmful to the child. While there remains no rigorous evidence that prenatal cannabis use causes offspring cognitive impairment, evidence of associations between prenatal cannabis use and preterm birth, low birthweight, small for gestational age, placental abruption, stillbirth, and admission to neonatal intensive care, continues to grow (Conner et al., 2015; Porath-Waller, 2015; Chabarria et al., 2016; The National Academies of Science Engineering and Medicine, 2017; Ko et al., 2018; Metz and Borgelt, 2018; Corsi et al., 2019; Luke et al., 2019), and should be an important consideration in discussions of cannabis use policy and recommendations. Suggesting that current policy is more harmful than prenatal cannabis use may create the false assumption among readers that current recommendations (Fried and Watkinson, 1990; Day et al., 1994) are unfounded and that prenatal cannabis use is safe. We maintain that no such evidence exists.

Associations between prenatal cannabis exposure and offspring cognitive functioning remain unclear. Broad cannabis legalization and the numerous limitations of the available evidence highlight an urgent need for high-quality, longitudinal studies that employ standardized measurement of cannabis exposure, have appropriate psychological assessment of cognitive outcomes, are adequately powered to detect minimally clinically significant differences in cognitive functioning while allowing the assessment and appropriate control of multiple confounding variables, and include appropriate adjustment for multiple statistical comparisons. We look forward to more research in this area, so the potential links between *in-utero* cannabis exposure and later cognitive functioning can be clarified and contribute to evidence-based recommendations regarding prenatal cannabis use.

## Author Contributions

KC, CL, and CM all contributed to the formation of concepts, composition, writing and substantive review, and editing of the manuscript. All authors approved the submission of the manuscript.

## Conflict of Interest

The authors declare that the research was conducted in the absence of any commercial or financial relationships that could be construed as a potential conflict of interest.
